# Plasma neutrophil gelatinase-associated lipocalin levels are associated with the presence and severity of coronary heart disease

**DOI:** 10.1371/journal.pone.0220841

**Published:** 2019-08-06

**Authors:** Chao Li, Zheng Zhang, Yu Peng, Hanxiang Gao, Yongxiang Wang, Jing Zhao, Chenliang Pan

**Affiliations:** 1 The First Clinical College of Lanzhou University, Lanzhou, Gansu, China; 2 Department of Cardiology, The First Hospital of Lanzhou University, Lanzhou, Gansu, China; 3 Gansu Key Laboratory of Cardiovascular Disease, Lanzhou, Gansu, China; Campus Bio-Medico University of Rome, ITALY

## Abstract

**Objective:**

This study aimed to compare the levels of plasma neutrophil gelatinase-associated lipocalin (NGAL), matrix metalloproteinase (MMP)-9, high-sensitivity C-reactive protein (hs-CRP), and interleukin (IL)-1β across different clinical presentations of coronary artery disease and to evaluate the relationship between those biomarkers and the severity of coronary artery lesions in patients without kidney disease.

**Methods:**

We examined 365 eligible patients who underwent coronary angiography. A total of 124 ST-segment elevation myocardial infarction (STEMI) patients, 117 stable angina pectoris (SAP) patients and 124 patients without atherosclerotic plaques were enrolled in the study. Plasma NGAL, MMP-9, hs-CRP, and IL-1β were measured in all patients using the enzyme-linked immunosorbent assay (ELISA) method. According to the SYNTAX score, the STEMI patients and SAP patients were divided into another set of 2 groups: a high score group (≥ 33, n = 29) and a low score group (<33, n = 212). The relationship between those biomarkers and the severity of coronary stenosis was examined by Spearman correlation analysis; the ability for NGAL to discriminate severe coronary stenosis was examined by receiver operating characteristic (ROC) curve; the influencing factors for the SYNTAX score were determined by logistic regression analysis.

**Results:**

Plasma NGAL, MMP-9, and hs-CRP levels in STEMI patients were higher than in the SAP patients and control subjects (P<0.05, respectively), and plasma NGAL and hs-CRP levels were significantly higher in the SAP patients than in control subjects (P<0.05, respectively), while plasma IL-1β was similar among the 3 groups (P>0.05, respectively). The SYNTAX score was positively related to NGAL (r = 0.363, P<0.001), MMP-9 (r = 0.377, P<0.001), and hs-CRP (r = 0.163, P<0.011); the SYNTAX score was not related to IL-1β (r = -0.043, P = 0.510). Plasma NGAL was positively related to MMP-9 (r = 0.601, P<0.001) and IL-1β (r = 0.159, P = 0.014). The area under the ROC curve for NGAL discriminating severe coronary stenosis was 0.838 (95% CI: 0.752–0.923, P<0.001), which was greater than that for MMP-9 [0.818, (95% CI: 0.724–0.912, P<0.001)], IL-1β [0.485, (95% CI: 0.369–0.601, P = 0.791)], and hs-CRP [0.607, (95% CI: 0.492–0.722, P = 0.061)]. Multivariate regression analysis showed that plasma NGAL levels were independently related to high SYNTAX scores [OR = 1.109, (95% CI: 1.104–1.114), P<0.001].

**Conclusion:**

Plasma NGAL, MMP-9, and hs-CRP levels in STEMI patients were higher than those in the SAP patients and control subjects. NGAL had a better ability to discriminate severe coronary stenosis than MMP-9, IL-1β, and hs-CRP. NGAL may be a novel biomarker to aid in risk stratification in coronary heart disease patients.

## Introduction

Inflammation plays a major role in atherosclerotic plaque destabilization. Acute myocardial infarction (AMI) is mainly due to atherosclerotic plaque rupture. Plaque rupture does not always cause an acute event; instead, healing of the plaque rupture results in stenosis progression [1, 2]. Neutrophil gelatinase-associated lipocalin (NGAL) has been extensively studied as a biomarker of acute kidney injury. Recent studies have shown that NGAL is also expressed in cardiomyocytes and macrophages in the atherosclerotic plaque and is bound with matrix metalloproteinase (MMP)-9[3, 4], thereby preventing MMP-9 degradation and reinforcing MMP-9 proteolytic activity, which can take part in the formation of an unstable atherosclerotic plaque[5, 6]. In the in vitro experiment, interleukin (IL)-1β strongly induced NGAL expression [7]. A previous study suggests that high-sensitivity C-reactive protein (hs-CRP) levels can reflect the stability of coronary atherosclerotic plaque [8]. However, the association between those biomarkers and the severity of coronary stenosis and those levels in patients with ST-segment elevation myocardial infarction (STEMI) compared with stable angina pectoris (SAP) is not yet clear. Thus, the purpose of this study was to compare the plasma NGAL, MMP-9, IL-1β, and hs-CRP levels in different clinical presentations of coronary heart disease (CHD) and to evaluate the relationship between those biomarkers and the severity of coronary stenosis in patients without kidney disease.

## Methods

### Study design and participants

This study was designed as a case-control study. We retrospectively collected data from 365 patients who had chest pain and underwent first-time coronary angiography (CAG) for suspected CHD at the Heart Center in the First Hospital of Lanzhou University between January 2014 and March 2017. This study adhered to the principles of the Declaration of Helsinki and was approved by the Ethics Committee of Lanzhou University First Hospital (NO. LDYYLL-2018-151). Written informed consent was obtained from all study participants.

A total of 365 patients without kidney disease were enrolled, and eligible patients were divided into 3 groups: STEMI group, n = 124; SAP group, n = 117; and control group, n = 124. Acute STEMI was diagnosed with the presence of chest pain for >30 min and < 12 h and persistent ST-segment elevation ≥ 2 mm in at least 2 contiguous precordial electrocardiography leads or ≥ 1 mm in at least 2 contiguous limb electrocardiography leads or a newly developed left bundle branch block [9]. SAP was defined as typical angina-like chest pain brought on by exertion and relieved by rest, sublingual nitrates, or both, a positive treadmill exercise test (>1 mm ST-segment depression) and stable obstructive lesion >50% in at least 1 of 3 major coronary arteries or major branches [10]. The control group was defined as patients who underwent coronary angiography for suspected CHD but were not found to have atherosclerotic plaques. Due to inpatients were enrolled, the control group did not include fully healthy volunteers. There were 58 patients with Hypertension, 11 patients with Diabetes Mellitus. The exclusion criteria were as follows: (1) patients with renal dysfunction (estimated glomerular filtration rate < 60 mL/min/1.73 m^2^ or on dialysis); (2) severe systolic ventricular dysfunction (left ventricular ejection fraction <30%); (3) history of heart failure or myocardial infarction; (3) acute or chronic inflammatory disease; (4) myocarditis; (5) significant valvular heart disease; (6) previous revascularization; (7) liver dysfunction; (8) pregnancy; (9) malignant tumors; and (10) incomplete clinical and laboratory data.

### Clinical parameters

CAG was performed according to standard techniques. SYNTAX scoring methods were used to evaluate the severity and lesion complexity of coronary artery disease. The images were assessed by two experienced cardiologists blinded to the study data. In the SYNTAX scoring system, each coronary lesion with a stenosis diameter of 50% or greater in vessels of 1.5 mm or more in diameter was scored. The latest online version (2.28) was used in the calculation of the SYNTAX scores (www.syntaxscore.com). According to the SYNTAX score, the STEMI and SAP patients were divided into another set of 2 groups: high SYNTAX score (≥33, n = 29) and low SYNTAX score (<33, n = 212) [11].

Baseline characteristics, including the presence of hypertension, diabetes mellitus, smoking status, family history of CHD, body mass index, lipid parameters, plasma creatinine, and blood cell counts, were collected from our databases. We defined hypertension as having a systolic/diastolic blood pressure > 140/90 mmHg on serial measurements or undergoing treatment with antihypertensive agents. Patients were considered to have type 2 diabetes mellitus if they had a fasting plasma glucose concentration ≥7 mmol/L, a nonfasting plasma glucose concentration ≥ 11.1 mmol/L, or were being treated with glucose-lowering drugs. The estimated glomerular filtration rate (eGFR) was calculated based on the Chinese equation that applies serum creatinine level, age, and sex as follows [12]:

$$\begin{array}{l} eGFR\;\left(ml/min/1.73\;m^{2}\right)\\ \;=175\times Cr\;{\left(mg/dl\right)}^{‐1.234}\times age^{‐0.179}\;\left(\times 0.79\;for\;female\;patients\right) \end{array}$$

### Laboratory methods

Blood samples were drawn from an antecubital vein before CAG and were collected in cold ethylenediaminetetraacetic acid (EDTA) tubes and centrifuged (2,000 g for 15 min) within 1 h, and plasma samples were stored in an −80°C freezer for subsequent analysis. Plasma NGAL, MMP-9, IL-1β, and hs-CRP levels were determined using stored EDTA-plasma with standard enzyme-linked immunosorbent assay (ELISA) using commercially available kits (CUSABIO, Wuhan, China) according to the manufacturer’s instructions.

Plasma NGAL concentrations were measured using the Human NGAL ELISA Kit. The measuring range of the kit was 0.0156–1.0 ng/ml, with an intra-assay coefficient variation of <8% and an inter-assay coefficient of variation of <10%. The minimum detection limit of the assay was 0.0039 ng/ml. Plasma MMP-9 concentrations were measured using the Human MMP-9 ELISA Kit. The measuring range of the kit was 0.312–20.0 ng/ml, with an intra-assay coefficient variation of <8% and an inter-assay coefficient of variation of <10%. The minimum detection limit of the assay was 0.284 ng/ml. Plasma IL-1β concentrations were measured using the Human IL-1β ELISA Kit. The measuring range of the kit was 125–8000 pg/ml, with an intra-assay coefficient variation of <8% and an inter-assay coefficient of variation of <10%. The minimum detection limit of the assay was 31.25 pg/ml. Plasma hs-CRP concentrations were measured using the Human hs-CRP ELISA Kit. The measuring range of the kit was 0.625–40.0 ng/ml, with an intra-assay coefficient variation of <8% and an inter-assay coefficient of variation of <10%. The minimum detection limit of the assay was 0.156 ng/ml. The detailed laboratory methods have been described in S1 File of the supporting information.

### Statistical analysis

Statistical analyses were performed with SPSS 22.0 (IBM, Armonk, NY, USA). The Kolmogorov-Smirnov test was used to test the distribution pattern. Continuous variables were presented as the mean ± standard deviation (SD) (normal distribution) or median with 25th and 75th percentiles (abnormal distribution). The continuous variables were compared using either Student’s t-test, an analysis of variance (ANOVA) (normal distribution), or the Mann-Whitney U or Kruskal-Wallis (abnormal distribution) tests. Categorical variables were summarized as frequencies with percentages and were compared using the chi-squared or Fisher test. Correlations between plasma levels of NGAL, MMP-9, hs-CRP, and IL-1β and the SYNTAX score were calculated by the nonparametric Spearman rank coefficient test. A receiver operating characteristic (ROC) curve analysis was carried out to document the predictive power of the plasma biomarker levels for discriminating high SYNTAX scores. The univariate and multivariate logistic regression analyses were used to calculate the influencing factors for the SYNTAX score. A two-tailed P-value less than 0.05 was considered statistically significant.

## Results

### Baseline clinical characteristics

Baseline characteristics are presented in Table 1. The smoking incidence, total cholesterol, low-density lipoprotein-cholesterol (LDL-C), white blood cell count, neutrophil, lymphocyte, and monocyte levels were significantly higher in the STEMI group than in the SAP and control groups (P< 0.05, respectively). Compared to the SAP group, the STEMI group showed a significantly higher SYNTAX score (P< 0.05). Nevertheless, the left ventricular ejection fraction (LVEF) and triglyceride levels were lower in the STEMI group than the SAP group (P< 0.05). There were no significant differences in age, sex, body mass index, hypertension, diabetes mellitus, family history of CHD, creatinine, eGFR, or platelets between the STEMI, SAP, and control groups (P> 0.05, respectively).

**Table 1:** Distribution of baseline characteristics in the study population.

Parameters	Control Group(n = 124)	SAP Group(n = 117)	STEMI Group (n = 124)	P value
Age (yr)	57.9±11.5	60.3±9.0	60.3±10.8	0.133
Men[n(%)]	90(72.6)	93(79.5)	99(79.8)	0.310
Body mass index(kg/m)	24.5(23.0–26.0)	25.0(23.0–27.0)	24.0(22.0–27.0)	0.427
Smoking[n(%)]	26(21.0)	32(27.4)	73(58.9)^a^^c^	<0.001
Hypertension[n(%)]	58(46.8)	62(53.0)	52(41.9)	0.227
Diabetes mellitus[n(%)]	11(8.9)	15(12.8)	19(15.3)	0.297
Family history of CHD[n(%)]	0	1(0.9)	2(1.6)	0.371
LVEF(%)	62.0(58.0–67.0)	64.0(57.5–70.0)	53.0(49.0–58.0) ^a^^c^	<0.001
Total cholesterol(mmol/L)	4.1±1.0	3.9±1.2	4.6±1.0^a^^c^	<0.001
LDL-C(mmol/L)	2.4±0.7	2.3±1.0	3.1±0.7^a^^c^	<0.001
HDL-C(mmol/L)	1.1±0.3	1.0±0.2^b^	1.1±0.2^a^	<0.01
Triglyceride(mmol/L)	1.3(1.0–1.8)	1.4(1.0–2.0)	1.2(0.8–1.7) ^a^	<0.05
Creatinine (um/L)	69.8±9.0	71.9±10.6	72.6±15.4	0.170
eGFR(mL/min/1.73 m^2^)	106.6±16.3	106.1±19.2	112.0±27.1	0.06
White blood cell count(×10^9^/L)	5.7±1.6	6.2±1.7	11.0±3.5^a^^c^	<0.001
Neutrophils(×10^9^/L)	3.5±1.3	3.8±1.5	8.9±3.5^a^^c^	<0.001
Platelets(×10^9^/L)	183.7±51.7	177.3±49.1	192.5±48.6	0.06
Lymphocytes(×10^9^/L)	1.6(1.4–2.1)	1.7(1.4–2.1)	1.4(1.0–1.8)^a^^c^	<0.001
Monocytes(×10^9^/L)	0.4(0.3–0.5)	0.4(0.3–0.5)	0.5(0.4–0.7)^a^^c^	<0.001
NGAL(ng/ml)	17.8(12.2–33.9)	41.5(28.7–58.5) ^b^	49.8(30.5–81.1)^a^^c^	<0.001
MMP-9(ng/ml)	243.1(175.3–330.8)	272.9(172.1–425.1)	815.1(459.6–1872.1)^a^^c^	<0.001
IL-1β(pg/ml)	29.9(19.8–44.2)	32.5(16.7–63.4)	30.5(20.5–62.8)	0.424
hs-CRP(mg/L)	0.8(0.5–1.7)	0.5(0.3–1.0) ^b^	1.8(1.3–3.7)^a^^c^	<0.001
SYNTAX score		18.0(9.0–45.5)	42.0(27.0–80.0)^a^	<0.001

STEMI: ST-segment elevation myocardial infarction; SAP: stable angina pectoris; NGAL: neutrophil gelatinase-associated lipocalin; MMP-9: matrix metalloproteinase-9; IL-1β: interleukin-1β; hs-CRP: high-sensitivity C-reactive protein; LVEF: left ventricular ejection fraction; eGFR: estimated glomerular filtration rate LDL-C: low-density lipoprotein-cholesterol; HDL-C: high-density lipoprotein-cholesterol.

a: STEMI Group vs SAP Group P<0.05

b: SAP Group vs Control Group P<0.05

c: STEMI Group vs Control Group P<0.05

Plasma NGAL, MMP-9, and hs-CRP levels in STEMI patients were higher than those in the SAP patients and control subjects (P< 0.05, respectively). Plasma NGAL and hs-CRP levels were significantly higher in the SAP group than in the control group (P< 0.05, respectively). Plasma IL-1β was similar among the groups (P> 0.05, respectively).

### Relationship between NGAL and MMP-9, IL-1β, and hs-CRP

**Fig 1:**
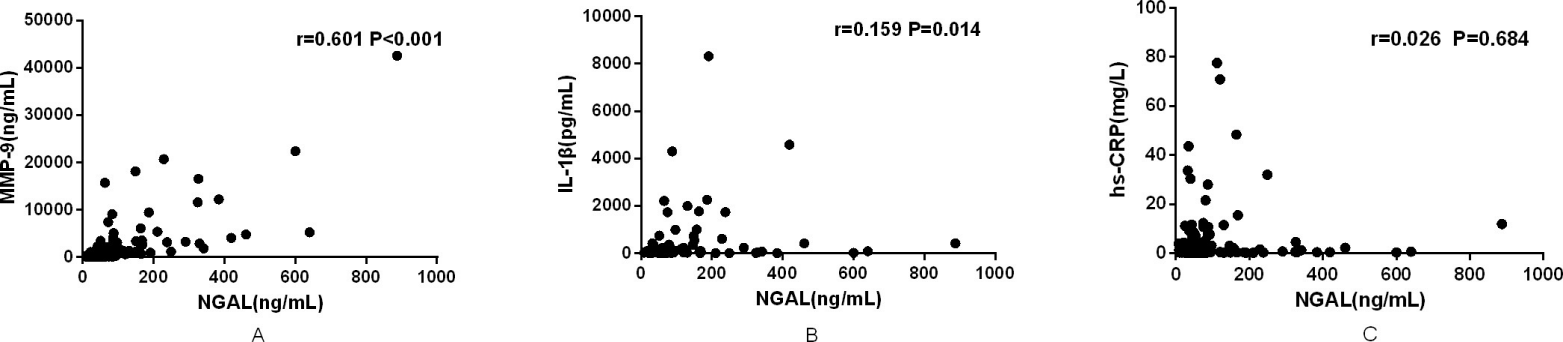
Relationship between NGAL, MMP-9, IL-1β, and hs-CRP. (A) Relationship between NGAL and MMP-9. (B) Relationship between NGAL and IL-1β. (C) Relationship between NGAL and hs-CRP. NGAL: neutrophil gelatinase-associated lipocalin; MMP-9: matrix metalloproteinase-9; IL-1β: interleukin-1β; hs-CRP: high-sensitivity C-reactive protein.

According to the Spearman rank correlation analysis, in all CHD patients, including STEMI and SAP patients, plasma NGAL was positively correlated with MMP-9 (r = 0.601, P<0.001) (Fig 1A) and IL-1β (r = 0.159, P = 0.014) (Fig 1B); however, there was no statistically significant correlation between NGAL and hs-CRP (r = 0.026, P = 0.684) (Fig 1C).

### Relationship between biomarkers and the SYNTAX score

**Fig 2:**
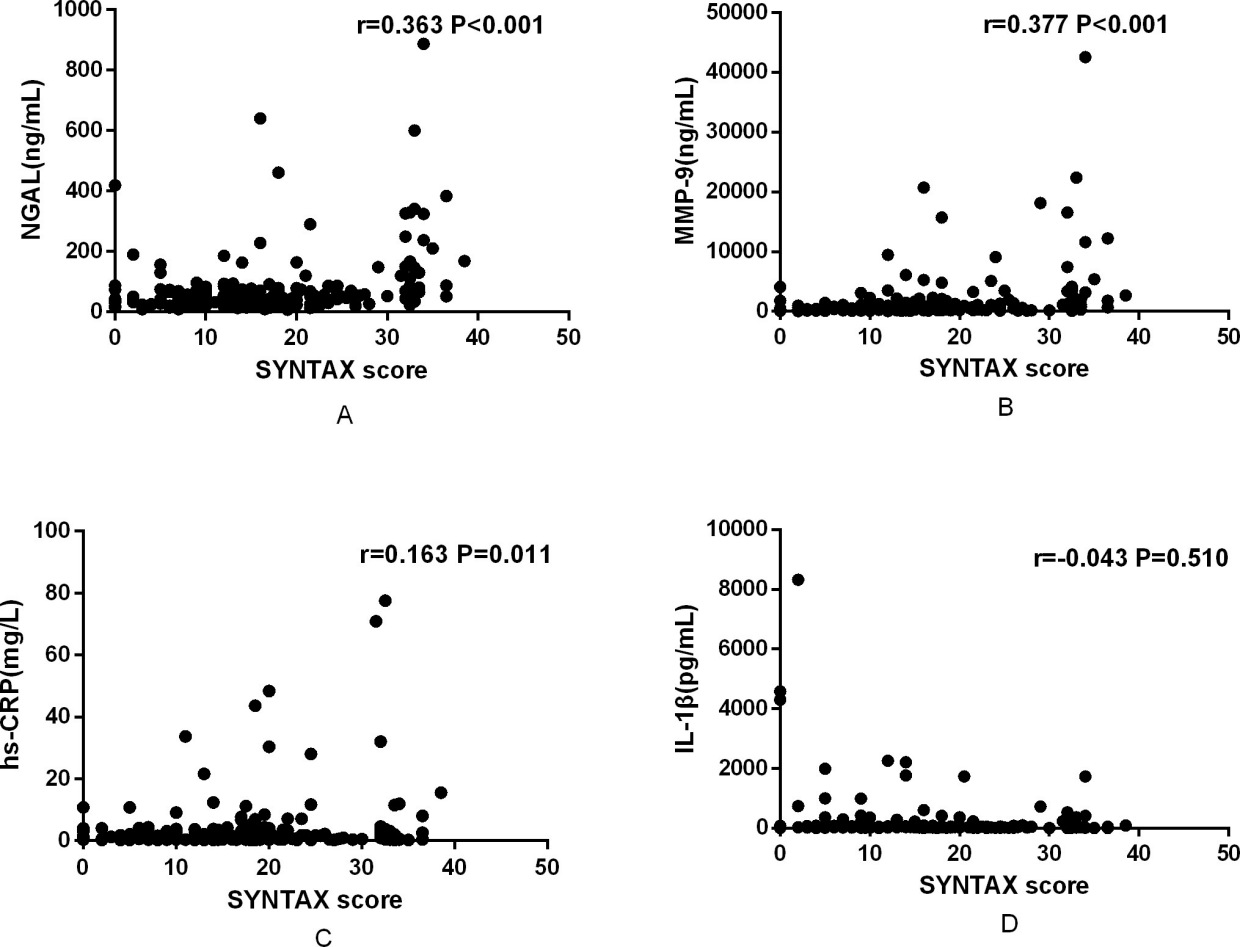
Relationship between biomarkers and SYNTAX score. (A) Relationship between SYNTAX score and NGAL. (B) Relationship between SYNTAX score and MMP-9. (C) Relationship between SYNTAX score and hs-CRP. (D) Relationship between SYNTAX score and IL-1β. NGAL: neutrophil gelatinase-associated lipocalin; MMP-9: matrix metalloproteinase-9; IL-1β: interleukin-1β; hs-CRP: high-sensitivity C-reactive protein.

To clarify the relationship between biomarkers and the SYNTAX score in CHD patients, we performed Spearman rank correlation analysis, which showed that the SYNTAX score was positively correlated with NGAL (r = 0.363, P<0.001) (Fig 2A), MMP-9 (r = 0.377, P<0.001) (Fig 2B) and hs-CRP (r = 0.163, P = 0.011) (Fig 2C), while SYNTAX score was not correlated with IL-1β (r = -0.043, P = 0.510) (Fig 2D).

### Plasma NGAL, MMP-9, IL-1β, and hs-CRP discriminating severe coronary stenosis

**Fig 3:**
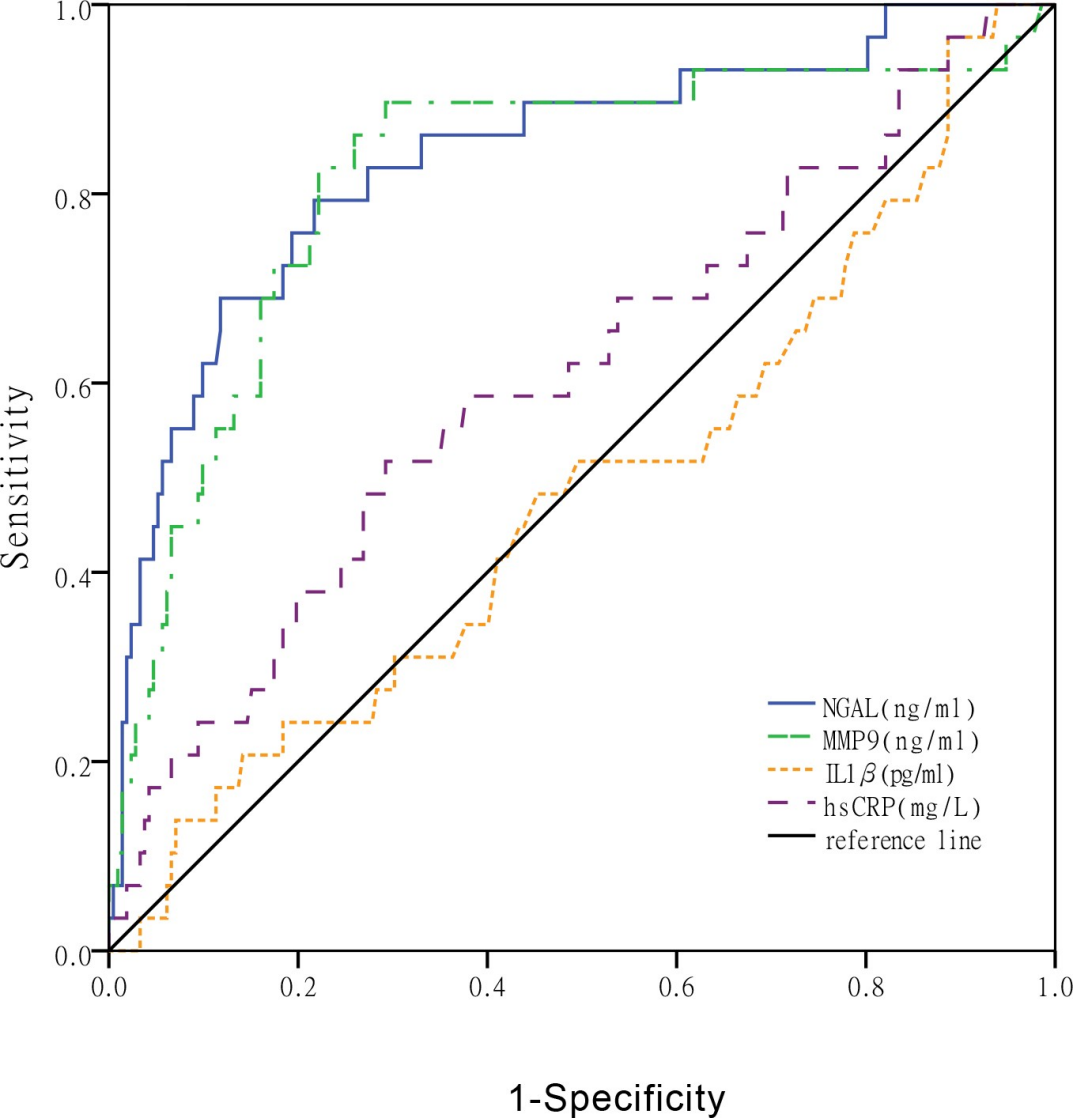
ROC curve performed for NGAL, MMP-9, IL-1β, and hs-CRP discriminating severe coronary stenosis.

The enrolled patients, including STEMI and SAP patients, were classified into two groups: a high SYNTAX score group (≥ 33, n = 29) and a low SYNTAX score group (< 33, n = 212). The area under the ROC curve for NGAL discriminating high SYNTAX score was 0.838 (95% CI: 0.752–0.923, P<0.001), which was greater than that for MMP-9 [0.818 (95% CI: 0.724–0.912, P<0.001)], IL-1β [0.485 (95% CI: 0.369–0.601, P = 0.791)], and hs-CRP [0.607 (95% CI: 0.492–0.722, P = 0.061)] (Fig 3).

To further explore the possible factors influencing the SYNTAX score, univariate and multivariate logistic regression analyses were performed (Table 2). Consequently, multivariate regression analysis indicated that the plasma NGAL level was independent of the high SYNTAX score [OR = 1.109 (95% CI: 1.104–1.114, P<0.001)].

**Table 2:** Logistic regression analysis of risk factors for the SYNTAX score.

Variables	Univariate		Multivariate	
	OR(95% Cl)	P	OR(95% Cl)	P
Smoking	1.996(0.908–4.389)	0.085	1.20(0.445–3.268)	0.714
LVEF	0.885(0.837–0.936)	<0.001	0.88(0.820–0.963)	0.004
Total cholesterol	1.530(1.108–2.113)	0.010	0.77(0.131–4.552)	0.774
LDL-C	1.691(1.136–2.517)	0.010	2.28(0.273–19.065)	0.446
HDL-C	10.988(2.063–58.539)	0.005	3.94(0.202–77.047)	0.365
White blood cell count	1.229(1.112–1.358)	<0.001	1.85(0.480–7.122)	0.371
Neutrophils	1.230(1.115–1.356)	<0.001	0.55(0.145–2.127)	0.391
Lymphocytes	0.454(0.214–0.963)	0.039	0.34(0.059–2.209)	0.240
Monocytes	4.589(1.357–15.513)	0.014	1.37(0.170–11.046)	0.767
NGAL	1.110(1.106–1.114))	<0.001	1.10(1.104–1.114)	<0.001
MMP-9	1.000(1.000–1.000)	<0.001	1.00(1.000–1.000)	0.310
hs-CRP	1.029(0.998–1.062)	0.065	1.00(0.963–1.040)	0.976

NGAL: Neutrophil gelatinase-associated lipocalin; MMP-9: matrix metalloproteinase-9; hs-CRP: high-sensitivity C-reactive protein; LVEF: left ventricular ejection fraction; LDL-C: low-density lipoprotein-cholesterol; HDL-C: high-density lipoprotein-cholesterol.

## Discussion

This study found that plasma NGAL, MMP-9, and hs-CRP levels in STEMI patients were higher than those in SAP and control patients, while plasma IL-1β was similar among the groups. Plasma NGAL levels were positively related with MMP-9 and IL-1β levels and severity of coronary stenosis.

Inflammation and dyslipidemia play a role in the pathogenesis of atherosclerosis and plaque destabilization. The primary cause of coronary artery thrombosis and STEMI are rupture of atherosclerotic plaques. Plaque rupture does not always cause an acute event either because of insufficient thrombus formation or a lumen area that is insufficiently flow limiting. Instead, healing of the plaque rupture leads to stenosis progression, and Hong et al. reported that 69% of patients with MI had culprit lesion plaque rupture versus 27% of those with SAP [1, 13]. Asymptomatic lesion progression of milder plaques might result from subclinical cycles of rupture and healing [2]. More recent studies have demonstrated that plaques progress from mild at baseline to obstructive at the time of MI, and plaque progression occurs before actual plaque rupture [2]. Our study found that total cholesterol, LDL-C, and some circulating inflammatory cells were higher in patients with STEMI compared with SAP patients and control subjects. Previous studies attributed an important role to neutrophils in the progression of atherosclerosis and acute coronary syndromes (ACSs) [14]. NGAL is a glycoprotein stored in granules of mature neutrophils and was initially isolated from neutrophils. Recent studies have shown that NGAL is also expressed in cardiomyocytes and macrophages in the atherosclerotic plaque [3, 4, 15]. In previous studies, which evaluated renal dysfunction and heart failure, CAG and percutaneous coronary intervention (PCI) were associated with increased plasma NGAL levels [16, 17]. Some studies have shown high plasma NGAL levels independently predicts all-cause mortality in STEMI patients treated with PCI independent of eGFR[18, 19], but recent research indicates that plasma NGAL levels do not predict mortality and cardiovascular disease risk independent of renal function and eGFR was the main determinant of plasma NGAL in patients with reduced eGFR (<60 ml/min) [20–22]. Thus, eligible patients included in this study did not have kidney disease or heart failure. Blood samples were taken before coronary angiography and PCI in our patients. Our study found that plasma NGAL and MMP-9 levels in STEMI patients were higher than those in SAP patients and control subjects, and plasma NGAL levels were significantly higher in the SAP group than the control group. In another study, Sahinarslan et al. found that plasma NGAL levels were higher in patients with AMI than in patients with stable coronary artery disease [23]. A previous study also confirmed that plasma MMP-9 levels were elevated in patients with AMI.

C-reactive protein is the most extensively investigated among the various inflammatory makers. It has been demonstrated that CRP and neutrophil leukocytes increase in the event of ACS and that neutrophils infiltrate atherosclerotic plaques and are involved in the inflammatory process leading to plaque rupture [18]. Our study also confirmed that plasma hs-CRP levels in STEMI patients were higher than those in SAP patients and control subjects and that plasma hs-CRP levels were significantly higher in the SAP group than in the control group. hs-CRP had a positive correlation with the severity of coronary artery lesions. Previous studies have reported that the hs-CRP level in the ACS patients was significantly higher than that in subjects in the healthy control group [24]. Their results showed that the plaque rupture observed in the high hs-CRP group (≥3 mg/L) was significantly more severe than that observed in the normal hs-CRP group (<3 mg/L). This suggests that hs-CRP levels can reflect the stability of coronary atherosclerotic plaques. Although some studies have confirmed a positive correlation between NGAL and CRP[25–27], it is interesting to note that this study confirms that there is no statistically significant correlation between plasma NGAL and hs-CRP, probably because our study excluded kidney disease and heart failure patients compared with previous studies. Consequently, there are differences in the degree of systemic inflammation and the main source of circulating NGAL in the different studies mentioned above. A previous study also found no correlation between serum NGAL and hs-CRP in coronary artery disease (CAD) patients without heart failure and chronic kidney disease [17]. Therefore, further research is needed to confirm the correlation between these two biomarkers.

Our study suggests that plasma IL-1β was similar among the groups. Due to objective conditions, the control group did not include fully healthy volunteers, and the small sample size we included resulted in no statistically significant data. IL-1β is an integral part of innate immune responses and is an early and prominent mediator of the inflammatory response in MI [28]. In our study, we found that plasma IL-1β levels were significantly and positively correlated with plasma NGAL levels. Previous studies have confirmed that IL-1β strongly induces NGAL expression in isolated neonatal cardiomyocytes [7]. This indicates a role for IL-1β in the regulation of NGAL expression in MI.

Previous studies have found that NGAL and MMP-9 are expressed in mouse models of MI and especially colocalized in areas with high proteolytic activity [4]. Boekhorst et al. found that NGAL is highly expressed in human atherosclerotic lesions and associated with increased MMP-9 activity [6]. We also found that plasma NGAL levels were significantly and positively correlated with plasma MMP-9 levels. MMP-9 is one of the endopeptidases that are expressed in vulnerable atherosclerotic plaques and is associated with plaque rupture. The main mechanism responsible for the inactivation of MMP-9 is inhibition by tissue inhibitor of metalloproteinase (TIMP-1), and the imbalance between proteolysis by MMP-9 and its inhibition by TIMP-1 may lead to plaque rupture [5]. NGAL is able to form a stable biologically active complex with MMP-9, increasing the MMP-9/TIMP-1 rate by inhibiting MMP-9 inactivation and leading to enhanced proteolytic activity with prolonged effects on collagen degradation[29]. NGAL expression levels are also associated with unstable plaque characteristics [29]. Thus, we conclude that high plasma NGAL levels are associated with the complexity and severity of atherosclerosis in CHD, and we certify that plasma NGAL levels significantly and positively correlate with the SYNTAX score. A previous study by Soylu et al. also found that plasma NGAL levels were associated with the SYNTAX score [30].

The SYNTAX score, an angiographic tool that is used to determine the complexity of coronary artery disease, takes into account not only the number of significant lesions and their locations but also the complexity and functional impact of angiographically obstructive lesions. Previous studies [11] have reported that a SYNTAX score cut-off value of ≥ 33 was considered relevant. Compared with coronary artery bypass grafting (CABG), mortality was significantly higher with a high SYNTAX score following PCI. Therefore, CABG should be recommended to CHD patients who have been allotted a high SYNTAX score. Our study suggests that plasma NGAL levels have a stronger discriminatory power than MMP-9, IL-1β, and hs-CRP for predicting a high SYNTAX score and are independently related to a high SYNTAX score. We further explored whether plasma NGAL was an independent risk factor for STEMI and SAP. Zografos et al. also found that NGAL was independently associated with the presence and severity of CHD in 73 patients undergoing coronary angiography [31]. Some recent prospective studies have confirmed that circulating NGAL levels are independently associated with long-term CHD outcomes in older women and significantly associated with all-cause mortality during long-term follow-up in ACS patients [32, 33]. Increased plasma NGAL levels after pPCI in STEMI patients were a better predictive marker of 30-day mortality than NGAL levels before pPCI [19]. These studies further confirmed the importance of NGAL in clinical applications. Interestingly, in contrast, a study showed that plasma NGAL levels do not predict the severity of CAD [34]. Other studies confirmed that plasma NGAL concentrations do not predict mortality independent of renal function and provide no additional benefit to inflammatory biomarkers and conventional cardiovascular risk factors in predicting cardiovascular events in stable CAD patients [20, 35]. The inconsistencies in the findings could be related to differences in sample sizes, population characteristics, or outcomes. Thus, in our study, we confirmed that plasma NGAL may be a biomarker for the severity of coronary stenosis and may help in risk stratification in patients. Further studies are needed to robustly evaluate circulating the potential utility of NGAL in predicting risk.

The present study has some limitations. First, because this is a retrospective single-center analysis, selection bias may not be entirely excluded. Second, due to the stringent inclusion criteria, the number of subjects was relatively small. Hence, a large-scale, multicenter, prospective study is necessary to confirm the results of this study. Of note, in our study, the NGAL concentrations in the control subjects, SAP, and STEMI patients are much lower than those reported in previous publications. These differences may be due to antibody differences in different kits, as the kits we studied were purchased from Chinese manufacturers. Although a very common ELISA test method was used, this difference has already occurred. Further research is needed to confirm this phenomenon.

## Conclusion

In conclusion, plasma NGAL, MMP-9, hs-CRP levels in STEMI patients were higher than those in the SAP patients and control subjects. NGAL had a better ability to discriminate severe coronary stenosis than MMP-9, IL-1β, or hs-CRP. NGAL is a novel biomarker that may aid in risk stratification in coronary heart disease patients.

## Supporting information

S1 File Supplemental methods. (DOCX)

S2 File Data set in this manuscript. (XLSX)
