# Supplementary material for: Plasma neutrophil gelatinase-associated lipocalin levels are associated with the presence and severity of coronary heart disease
Source: PLoS One. 2019 Aug 6;14(8):e0220841. doi: 10.1371/journal.pone.0220841 (PMC6684288; doi:10.1371/journal.pone.0220841)
Supplement: S1 File — (DOCX) [file pone.0220841.s001.docx]

**S1 File**

**Supplemental Methods**

Blood samples were drawn from an antecubital vein before CAG and were collected in cold ethylenediaminetetraacetic acid (EDTA) tubes and centrifuged (2,000 g for 15 min) within 1 h, and plasma samples were stored in an −80°C freezer for subsequent analysis. Plasma NGAL, MMP-9, IL-1β, and hs-CRP levels were determined using stored EDTA-plasma with standard enzyme-linked immunosorbent assay (ELISA) using commercially available kits (CUSABIO, Wuhan, China).

Plasma NGAL concentrations were measured using the Human NGAL ELISA Kit. This assay employs the quantitative sandwich enzyme immunoassay technique. The measuring range of the kit was 0.0156-1.0 ng/ml, with an intra-assay coefficient variation of <8% and an inter-assay coefficient of variation of <10%. The minimum detection limit of the assay was 0.0039 ng/ml. Plasma samples from 365 eligible patients were diluted 1:400. A volume of 100 μl each of the standard (provided by the manufacturer), sample (STEMI, SAP, and control plasma sample), or sample diluent alone (blank) was rapidly added to a 96-well plate precoated with NGAL antibody and incubated at 37ᵒC for 2 h. The liquid was removed from each well, and the wells were not washed. Thereafter, 100 μl of biotin-antibody (1x) working solution was added to each well and incubated at 37ᵒC for 1 h. After washing (3 times), 100 μl of avidin-conjugated horseradish peroxidase (1x) working solution was added to each well and incubated at 37ᵒC for 1 h. After washing (5 times), 90 μl of the chromogenic peroxidase substrate tetramethylbenzidine was added to each well. The plate was incubated for 30 min at 37ᵒC (protected from light). Then, the reaction was stopped by adding 50 μl of ‘‘stop solution’’, and the absorbance at 450 nm was read with a microplate reader. The professional software "Curve Expert", downloaded from CUSABIO's web, was used to make a standard curve. The concentration read from the standard curve must be multiplied by the dilution factor.

Plasma MMP-9 concentrations were measured using the Human MMP-9 ELISA Kit. This assay employs the quantitative sandwich enzyme immunoassay technique. The measuring range of the kit was 0.312-20.0 ng/ml, with an intra-assay coefficient variation of <8% and an inter-assay coefficient of variation of <10%. The minimum detection limit of the assay was 0.284 ng/ml. Plasma samples from 365 eligible patients were diluted 1:100. A volume of 100 μl each of the standard (provided by the manufacturer), sample (STEMI, SAP, and control plasma sample), or sample diluent alone (blank) was rapidly added to a 96-well plate precoated with MMP-9 antibody and incubated at 37ᵒC for 2 h. The liquid was removed from each well, and the wells were not washed. Thereafter, 100 μl of biotin-antibody (1x) working solution was added to each well and incubated at 37ᵒC for 1 h. After washing (3 times), 100 μl of avidin-conjugated horseradish peroxidase (1x) working solution was added to each well and incubated at 37ᵒC for 1 h. After washing (5 times), 90 μl of the chromogenic peroxidase substrate tetramethylbenzidine was added to each well. The plate was incubated for 20 min at 37ᵒC (protected from light). Then, the reaction was stopped by adding 50 μl of ‘‘stop solution’’, and the absorbance at 450 nm was read with a microplate reader. The professional software "Curve Expert", downloaded from CUSABIO's web, was used to make a standard curve. The concentration read from the standard curve must be multiplied by the dilution factor.

Plasma IL-1β concentrations were measured using the Human IL-1β ELISA Kit. This assay employs the quantitative sandwich enzyme immunoassay technique. The measuring range of the kit was 125-8000 pg/ml, with an intra-assay coefficient variation of <8% and an inter-assay coefficient of variation of <10%. The minimum detection limit of the assay was 31.25 pg/ml. Plasma samples from 365 eligible patients were not diluted. A volume of 100 μl each of the standard (provided by the manufacturer), sample (STEMI, SAP, and control plasma sample), or sample diluent alone (blank) was rapidly added to a 96-well plate precoated with NGAL antibody and incubated at 37ᵒC for 2 h. The liquid was removed from each well, and the wells were not washed. Thereafter, 100 μl of biotin-antibody (1x) working solution was added to each well and incubated at 37ᵒC for 1 h. After washing (3 times), 100 μl of avidin-conjugated horseradish peroxidase (1x) working solution was added to each well and incubated at 37ᵒC for 1 h. After washing (5 times), 90 μl of the chromogenic peroxidase substrate tetramethylbenzidine was added to each well. The plate was incubated for 30 min at 37ᵒC (protected from light). Then, the reaction was stopped by adding 50 μl of ‘‘stop solution’’, and the absorbance at 450 nm was read with a microplate reader. The professional software "Curve Expert", downloaded from CUSABIO's web, was used to make a standard curve. The concentration read from the standard curve must be multiplied by the dilution factor.

Plasma hs-CRP concentrations were measured using the Human hs-CRP ELISA Kit. This assay employs the quantitative sandwich enzyme immunoassay technique. The measuring range of the kit was 0.625-40.0 ng/ml, with an intra-assay coefficient variation of <8% and an inter-assay coefficient of variation of <10%. The minimum detection limit of the assay was 0.156 ng/ml. Plasma samples from 365 eligible patients were diluted 1:1000. A volume of 100 μl each of the standard (provided by the manufacturer), sample (STEMI, SAP, and control plasma sample), or sample diluent alone (blank) was rapidly added to a 96-well plate precoated with NGAL antibody and incubated at 37ᵒC for 1 h. The liquid was removed from each well. After washing (3 times), 100 μl of avidin-conjugated horseradish peroxidase (1x) working solution was added to each well and incubated at 37ᵒC for 1 h. After washing (5 times), 90 μl of the chromogenic peroxidase substrate tetramethylbenzidine was added to each well. The plate was incubated for 20 min at 37ᵒC (protected from light). Then, the reaction was stopped by adding 50 μl of ‘‘stop solution’’, and the absorbance at 450 nm was read with a microplate reader. The professional software "Curve Expert", downloaded from CUSABIO's web, was used to make a standard curve. The concentration read from the standard curve must be multiplied by the dilution factor.
